# Properties and Performance of Novel Mg(OH)_2_-Based Coatings for Corrosion Mitigation in Concrete Sewer Pipes

**DOI:** 10.3390/ma13225291

**Published:** 2020-11-23

**Authors:** Domna Merachtsaki, Georgios Fytianos, Efthimios Papastergiadis, Petros Samaras, Haris Yiannoulakis, Anastasios Zouboulis

**Affiliations:** 1 Division of Chemical and Environmental Technology, Department of Chemistry, Aristotle University of Thessaloniki, 54124 Thessaloniki, Greece; meradomn@chem.auth.gr; 2 Department of Food Science and Technology, International Hellenic University, 57400 Thessaloniki, Greece; gfytianos@gmail.com (G.F.); efspa@food.teithe.gr (E.P.); samaras@ihu.gr (P.S.); 3 Research and Development Centre, Grecian Magnesite S.A., 57006 Thessaloniki, Greece; h.giannoulakis@grecianmagnesite.com

**Keywords:** concrete protection, corrosion control against sulfuric acid, sewerage pipe systems, magnesium hydroxide coatings, cellulose

## Abstract

The biological activity occurring in urban sewerage systems usually leads to the (biogenic) corrosion of pipe infrastructure. Anti-corrosion coating technology was developed in an effort to protect sewer pipes from degradation. This study evaluates a new class of relatively low-cost magnesium hydroxide-based coatings, regarding their ability to adhere efficiently onto the concrete surface, and offer efficient corrosion protection. Six magnesium hydroxide-based coatings were prepared with the addition of two different types of cellulose, used as adhesion additives, and these were applied on concrete specimens. Pull-off measurements showed that the addition of higher amounts of cellulose could improve the coating adhesion onto the concrete surface. An accelerated sulfuric acid spraying test was used to evaluate the consumption time of the applied coatings and their efficiency in maintaining over time slightly alkaline pH values (above 8) on the coated/protected surfaces. At the end of spraying test, a mineralogical analysis of surface samples was performed, indicating that the formation of corrosion by-products (mainly gypsum) was increased when the added amount of cellulose was lower. Hardness and roughness measurements were also conducted on the concrete surfaces, revealing that the coatings helped the concrete surface to preserve its initial surface properties, in comparison to the uncoated specimens. A SEM/microstructure analysis showed that aggregates were formed (possibly consisting of Mg(OH)_2_), affecting the reactivity of the protected surface against sulfuric acid attack.

## 1. Highlights

Application of magnesium hydroxide-based coatings for the mitigation of corrosion in concrete sewer pipes.

The addition of 1% cellulose (wt. of solids) in the applied coating showed great adhesion ability, and water and acid resistance properties.

## 2. Introduction

**Figure 1:**
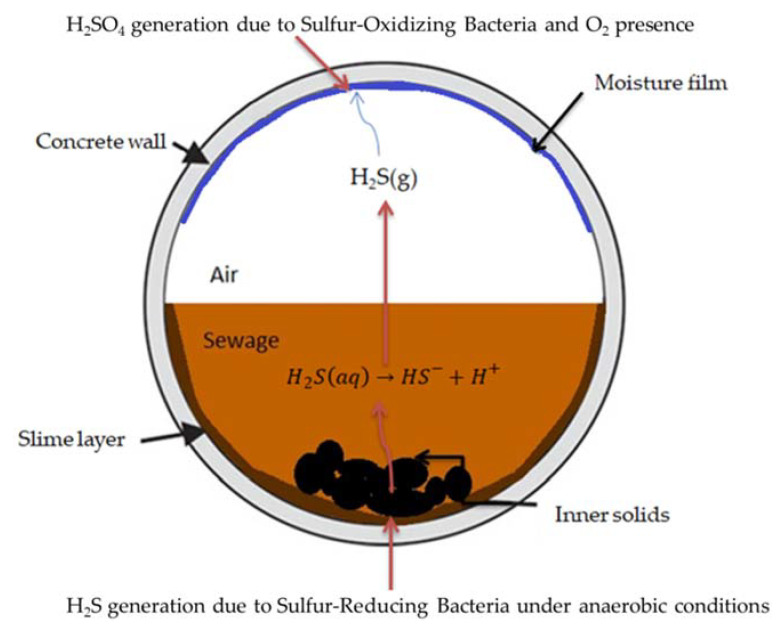
Cross-section representation of a concrete sewer pipe. Reproduced from Ref. [5] 2020, MDPI.

Microbiologically induced corrosion (MIC) or bio-corrosion in concrete sewerage systems, especially considering those with larger diameters, is a known problem [1], involving the generation of hydrogen sulfide (H_2_S). This dangerous gas originates in the submerged portion of sanitary pipe sewers, where anaerobic conditions generally prevail, due to the presence of sulphur-reducing bacteria (SRB) located in the slime layer, i.e., the layer consisting of bacteria and inert solids, commonly existing in the flooded part of sewer pipes. The H_2_S (gas) subsequently can be diffused towards the upper (empty, “atmospheric”) part of the sewer pipe (usually termed as the “crown”) above the flowing wastewater surface, lowering the surface pH of exposed concrete walls/surfaces and favoring the development of sulfur-oxidizing bacteria (SOB). As a result, biogenic H_2_SO_4_ is ultimately produced, corroding the inner surface of concrete sewer pipes by reacting with the hydrated cement paste, and eventually producing (unwanted) gypsum, leading to the collapse of pipes [2,3]. In the sewerage network, wherever there is an adequate presence of H_2_S gas (e.g., >2 mg/L), high relative humidity, and rather high atmospheric oxygen content, MIC can take place. These conditions are commonly existent in the majority of wastewater transferring systems, at least several times yearly, depending also on seasonal parameters (e.g., influencing the wastewater flow rate) [4]. The intensity and extent of MIC depends mainly on the specific configuration and characteristics of each individual sewerage network. Figure 1 presents a relevant representation from a typical concrete sewer pipe section [5]. It is a fact that the components existing in the sewerage system affect hydrogen sulfide production and microbial activity, which are indirectly responsible for the biogenic sulfuric acid production, but the main corrosive agent in MIC is considered to be the sulfuric acid. As a result, several researchers have focused on the study of concrete corrosion, due particularly to biogenic sulfuric acid. The respective procedures may take place in situ (i.e., in the sewer) [6], or under controlled laboratory conditions [7], using the respective chemical agent (sulfuric acid) [8], or by developing microorganisms that can produce the biogenic sulfuric acid (e.g., in the presence of hydrogen sulfide) [9]. In the present study the chemical sulfuric acid was used for the accelerated experimental tests, simulating the existing conditions in a sewer pipe.

The economic losses due to MIC are considered to be very high worldwide [10]. It is estimated that the respective annual pipe rehabilitation costs may reach over $400 million in Germany and over $100 million in the United Kingdom [11], depending also on other parameters (e.g., network age, maintenance schedule, etc.). In Flanders (Belgium) the MIC related costs are approximately 10% of the total sewage treatment cost [12]. It should also be noted that it is sometimes very difficult and costly for the municipalities to repair the old and deteriorated concrete sewer pipes, especially those of larger cross sections, since the sewers can be located under historic and densely populated places [13]. Today, despite the fact that the material of choice to be used for the construction of new sewerage systems is a whole new research field, providing several alternative solutions, such as the concrete incorporating polymeric substances [7], there are still numerous cement and concrete sewerage pipe systems in operation.

The various MIC mitigation strategies commonly applied, can be categorized into three main groups, i.e., (i) corrosion resistant concrete materials [7,14], (ii) protective coatings [13,15], and (iii) chemical dosing of specific reagents directly into the transferred wastewater [16,17]. The use of Mg(OH)_2_ has been previously studied as a neutralizing agent for acidic wastes [18], as well as a protective coating [19]. The present study focuses on the protection of concrete pipes from corrosion, by applying relatively low-cost Mg(OH)_2_-based coatings and by using common adhesion additives (celluloses). The spraying of the concrete pipes with an appropriate Mg(OH)_2_-based slurry could be a sustainable and economic solution for their protection from corrosion and it is considered among the most cost-effective strategies to mitigate MIC problems, especially in Greece [20].

Limited research has been performed so far regarding the evaluation of Mg(OH)_2_-based consumption of coatings due to the reaction with sulfuric acid through accelerated tests on concrete specimens [19,21]. The relevant studies have mainly focused on the preservation of alkaline surface pH values using Mg(OH)_2_ coatings onto the concrete surfaces. However, the adhesion of the respective coatings onto the concrete surface is a crucial property that also needs to be examined carefully. There are many factors that can be studied, in order to evaluate the performance of coatings and the protection of the concrete’s surface against corrosion/degradation problems, such as the adhesion ability [22], the thickness change [23], the surface characteristics [13], the concrete properties [24], the mass change [25], etc. In this study the factors selected to be examined are the adhesion properties of the coating, the water and acid resistance, the mineralogical changes, the change in roughness and hardness of the concrete substrate after acid spraying, and the surface characteristics of the coatings. The purpose of this study is to improve (and evaluate) the adhesion ability of the relatively low-cost Mg(OH)_2_-based coatings, by using methyl-cellulose and/or carboxymethyl-cellulose as common adhesion additives. At the same time, this research evaluates the corrosion protection that these coatings can offer to the concrete surface, elucidating the respective coating–protection mechanisms. The amount of adhesive additives, influencing the ability for corrosion protection of the coating, was studied by monitoring the changes in the existing mineralogical phases. These changes refer mainly to the consumption of brucite (Mg(OH)_2_) and the formation of gypsum (CaSO_4_). The major characteristics and the different physicochemical properties of the six different coatings presented, selected among several others as bio-corrosion inhibitors, were evaluated by using different material characterization techniques. The ultimate aim of this paper was to determine the optimum cellulose combination and cellulose amount to be added in the Mg(OH)_2_-based coatings, and to examine the various important application properties, such as adhesion, water and acid resistance, and corrosion protection ability.

## 3. Materials and Methods

### 3.1. Concrete Specimens Preparation

The specific concrete type used in the experiments was selected in order to appropriately simulate the respective concrete that is commonly used for the construction of sewerage pipes. According to the EN 1766:2000 Standard [26] and the BS EN 196-1:1995 Standard [27] the concrete type MC 0.45 was selected. The components used for the production of these specimens were cement type CEM I 42.5 R, limestone sand 0–4 mm and limestone grit 4–8 mm (applied as natural aggregates), tap water, and a Sika Viscocrete superplasticizer. A water/cement ratio of 0.45 was applied. The fresh concrete was poured into wooden molds, and the air from the concrete mixture was removed by using a vibration table. Concrete samples of different dimensions were prepared, depending on the respective applicable testing procedures (i.e., 200 mm × 200 mm × 20 mm, 50 mm × 50 mm × 20 mm and cylindrical/diameter 30 mm × height 60 mm). After 24 h the specimens were de-molded and cured in water for 27 days at 20 ± 2 °C. After curing, the concrete samples were stored under normal laboratory conditions at temperature of 21 ± 2 °C and relative humidity 60% ± 10%.

### 3.2. Surface Coatings

The examined coatings were prepared by using a Mg(OH)_2_ powder (MH), supplied by Grecian Magnesite S.A. (Gerakini, Greece). As adhesion promoters onto the concrete surface, methyl-cellulose (MC) (Mecellose FMC 23007, Samsung Fine Chemicals) (Samsung Fine Chemicals, Hessen, Germany) and carboxymethyl-cellulose (CMC) (Optapix C 12, Zschimmer&Schwarz GmbH & Co KG) (Burgstädt, Germany) were used. Deionized water was used for all coating preparations, resulting in slurries with 57.5% wt. content of solids. Dispersant polymer for mineral slurries (Acumer 9300), supplied by Dow (Midland, MI, USA) was added as a stabilizer for the slurries, and as viscosity reducer at 0.6% wt. of solids. Table 1 shows the typical composition of the raw material used (MH).

Based upon preliminary experiments, two series of magnesium hydroxide slurries were synthesized by using methyl-cellulose as an adhesive in three different concentrations, i.e., 1%, 0.4%, and 0.1% wt. content of solids (the respective samples were termed as M1m, M2m, and M3m). The second series was prepared by using the combination of methyl-cellulose and carboxymethyl-cellulose at 1%, 0.4%, and 0.1% wt. content of solids of a mixture of both celluloses (termed as M1mc, M2mc, and M3mc, respectively). The celluloses used in the second series of slurries were mixed in the ratio MC/CMC = 4/1.

Deionized water was heated at 90 °C and then Acumer 9300 and celluloses were added into the hot water, under stirring until the solution reached room temperature. Magnesium hydroxide powder was added subsequently under mechanical stirring for 1 h, until the slurry become homogenous.

### 3.3. Application of the Coatings

The fast drying of applied coatings may lead to crack formation. In order to avoid this, the concrete surface was moistened with deionized water before the coating application. A spatula was used for the application of the coatings on the concrete specimens, in a way that a uniform thickness of the coating could be achieved. The coatings were applied on concrete blocks, in two different amounts (thickness). The amount of the applied coating was selected to be 0.0008–0.001 g/mm^2^ and 0.0018–0.002 g/mm^2^ for the thin and the thick layer, respectively, according to our previous relevant experience and the respective preliminary experiments. The thickness of protective coatings may vary, depending on the various kinds of materials that can be used for this purpose. The organic coatings, usually applied for the protection of concrete surfaces, can be commonly applied with thicknesses between 0.1–1 mm; whereas the cementitious coatings and linings used on concrete surfaces may be applied at higher thickness, i.e., up to few a mm [28]. The thickness of polymer-modified cementitious coatings was 2 mm in a relevant study [28], while another study used epoxy coatings with thicknesses ranging between 0.3–0.4 mm, and modified cement mortar coatings with thicknesses ranging between 2–3 mm [29]. The amounts of coating applied in this study were appropriately selected in order to achieve thicknesses between 0.5–1.5 mm, i.e., intermediate between the two aforementioned cases, since the examined materials are neither strictly polymeric, nor cementitious. After the application of the coating the coated concrete samples were allowed to dry under normal laboratory conditions (temperature 21 ± 2 °C and relative humidity 60% ± 10%), for 3 days before testing.

### 3.4. Coating Adhesion on Concrete

The adhesion of the coatings onto the concrete substrate was studied by using the pull-off bond testing method. The test procedure was based on the respective standards, EN 1542:1999 [30] and EN 13578:2003 [31]. Concrete specimens with dimensions 200 mm × 200 mm × 20 mm were used and drilled with a diamond-coring barrel to a depth of 5 mm into the concrete substrate. In that way cylindrical channels (with depth 5 mm) were created on the concrete surface, surrounding the cyclical concrete surface to be coated. The coatings were applied with two different thicknesses, as described in the previous Section 3.3, on the cyclical concrete surfaces, surrounded by the channels. Specific caution should be taken so that the coating remains on the concrete surface, and is not inserting into the surrounding channels. The coated specimens were stored under normal laboratory conditions (i.e., temperature 21 ± 2 °C and relative humidity 60% ± 10%), for 3 days before testing in order to be dried. According to the aforementioned standards an adhesive (SINMAST P103, Sintecno, Koropi, Greece) was then applied on the coatings’ surface, and the dollies were placed on the core face. Dollies are metallic cylinders that can be adjusted on the pull-off equipment, so that the measurement can appropriately be conducted. The pull-off equipment is used to apply the load initially on the dollies and subsequently on the coating, aiming to cause the tensile bond failure of the specimen. After the supplementary adhesive hardened, the failure load was measured by using the pull-off equipment (Digital pull-off strength tester, Matest) (Matest, Italy) and recorded.

### 3.5. Water and Acid Resistance

**Figure 2:**
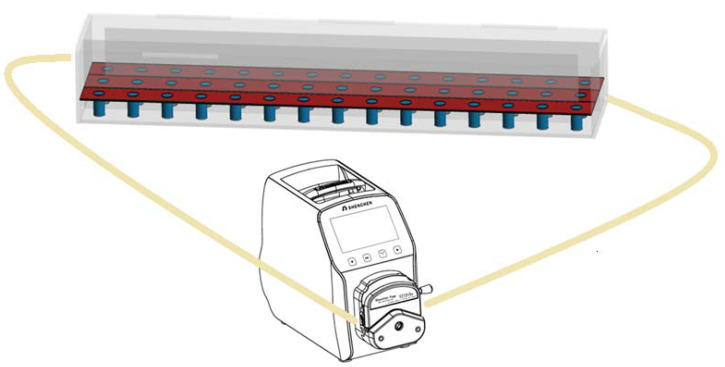
Experimental device for studying the concrete specimens’ deterioration after coating, due to the presence of H_2_SO_4_ (0.2 M) in comparison with water. The acrylic glass device allows the circulation of solution through an S-shaped route.

Coated cylindrical concrete specimens (with diameter 30 mm and height 60 mm) were immersed in a custom-made device (140 cm × 30 cm × 20 cm) in order to examine the adhesion properties of the examined coatings under the influence of different environments. The acrylic glass device was divided into 3 rows, allowing for the placement of 15 samples per row (i.e., totally 45 specimens can be simultaneously examined). After the liquid phase (water or sulfuric acid) passes through the first row, an opening for the flow enables it to pass through the second row and then to the third row, following an S-shaped route from the entry point to the final outlet and then, back to the pump. The coated specimens were immersed in water, as well as in 0.2 M sulfuric acid solution. The experimental device allowed the circulation of the liquid phase by the use of a peristaltic pump, applying an adjustable flow rate, corresponding to horizontal velocity equal to 0.05 m/s (Figure 2).

In a combined sewage collection system of concrete sewer pipes, surface (mainly rain) runoff and sewage are simultaneously collected. The upper part of the concrete sewer pipes, which will be covered with the Mg-based coating, may be in direct contact with the water, e.g., in the case of sudden storm flushing, while during the dry periods wastewater flow is reduced, eliminating the contact of water with the applied coating. That is the reason why the authors have examined the water resistance properties of the coatings. Moreover, although several components can be transferred with the sewage along the sewer pipes, the authors focused their attention on the influence of sulfuric acid, since the biogenic-produced sulfuric acid is considered as the main corrosive agent inside the sewer pipe [7].

### 3.6. Coating Performance under High Velocities

**Figure 3:**
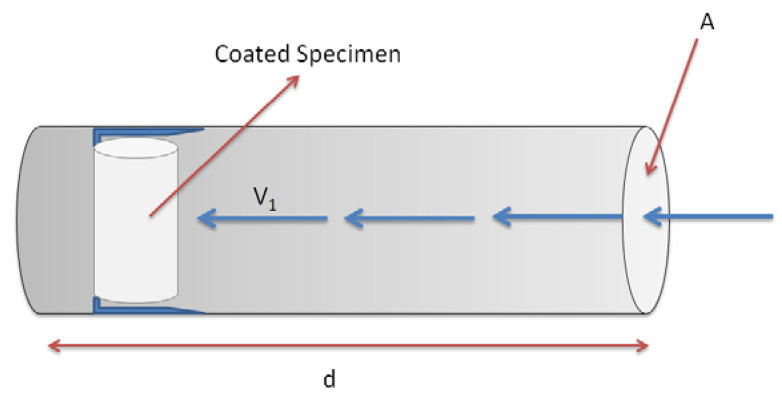
Experimental device for studying the effect of higher than critical solution velocities on the adhesion of coatings in concrete specimens.

In addition to pull-off tests, supplementary tests were also carried out under high horizontal-velocity of the examined solution in order to examine the performance of coatings under rather extreme conditions of water flow (in order to simulate the sewerage network performance during a heavy storm incident in a combined sewer system). The concrete specimens were placed into a custom-made laboratory device (Figure 3) and high flow velocities (i.e., V_1_ = 14 m/s) were applied on the specimen surfaces for the duration of 20 min. The plastic tube (60 mm diameter) had two protrusions near the end, allowing the placement of one specimen at a time. However, for comparison reasons it should be noted that the maximum velocity of transferred wastewater in cement pipelines does not usually exceed 3 m/s [32]. This experiment was carried out additionally to the pull-off test in order to examine the adhesion of coatings under high velocities, which normally exceed the critical velocities of sewerage systems.

### 3.7. Acid Spraying

This experiment was an accelerated procedure in order to evaluate the coatings’ consumption and protection ability, considering the real conditions existing in sewer pipes, which over time permit the application of much larger contact/reaction times.

A concentrated H_2_SO_4_ solution (4 M) was used and sprayed with an appropriate handheld spraying device on the coated concrete specimens. The coatings’ mass that theoretically can react with the sulfuric acid was calculated, according to the respective magnesium and calcium content, when the later were expressed as oxides. The theoretically expected time of the coatings’ total consumption (i.e., 100% reaction stoichiometry) was selected to be 6 days, according to previous experience, and based on preliminary relevant experiments and stoichiometric titrations. The total mass of the required acid solution for daily spraying was accordingly calculated, in order to achieve the daily consumption of a specifically pre-determined amount of coating, based upon the theoretical amount (see also the Appendix A for detailed information, regarding this calculation). This corresponded to 17% of the respective reaction stoichiometry for each day of spraying, in order to achieve the 100% reaction stoichiometry at the end of the 6 days experiment, and the procedure was performed by adjusting the number of daily spraying applications accordingly.

**Figure 4:**
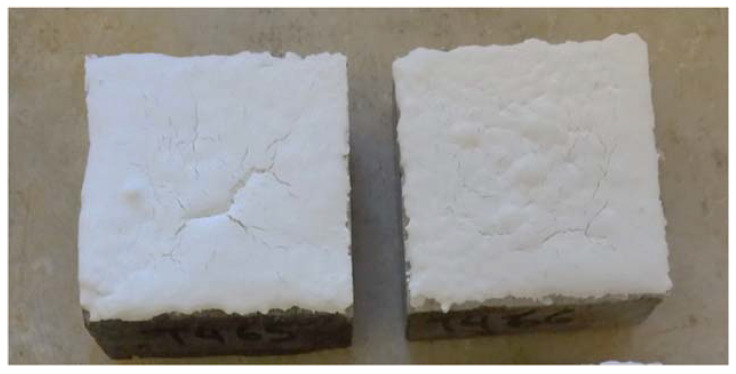
Concrete blocks (specimens) coated on one side (50 × 50 mm) by the M1cm sample.

Concrete specimens with dimensions 50 mm × 50 mm × 20 mm were used for the (accelerated) sulfuric acid spraying tests. The coatings were applied on one side (50 mm × 50 mm) of the concrete blocks (Figure 4). Each day the coated specimens were sprayed multiple times, allowing the (pre-calculated) sulfuric acid mass to react with the corresponding amount of coating on the specimens’ surface.

The surface pH of coatings was measured daily with a flat surface pH electrode (Extech PH100: Waterproof ExStik pH meter, Extech Instruments) (Extech Instruments, Nashua, NH, USA). All measured pH values of the samples were recorded before spraying as the starting values. The specific spots to be measured were wetted with 1 mL of deionized water prior to the measurement. For comparison reasons, uncoated concrete specimens were also sprayed with sulfuric acid and measured. A climatic test chamber was used for the storage of samples throughout the duration of acid spraying experiments, simulating appropriately the conditions within the sewer pipes at the temperature of 20 ± 2 °C and 99% relative humidity.

The spraying testing procedure was terminated when the respective coating was consumed, or when the extended formation of gypsum (a commonly formed by-product) was optically observed. The total consumption/reaction of coatings with the added sulfuric acid solution could not be achieved, due to the gypsum formation, which was initiated even when a small area of the concrete surface would be exposed to sulfuric acid (i.e., due to the coating’s consumption). Gypsum is the product of the reaction between sulfuric acid and concrete constituents, and indicates that the applied coating cannot longer sufficiently protect the concrete surface.

### 3.8. Roughness and Hardness

The degradation of concrete affects the concrete’s fundamental properties. The respective hardness and roughness measurements of the concrete surface can indicate the degree of protection that the applied coatings may provide to the concrete. The measurements of surface roughness and hardness were both conducted on uncoated specimens, as well as on sprayed-coated specimens. The surface roughness was measured with an electronic portable roughness gauge (Rugosurf 20, Tesa) (TESA Technology, North Kingstown, RI, USA), while the hardness measurements were carried out by a Shore A, hardness tester (Sauter) (Sauter GmbH, Balingen, Germany). Roughness was expressed in Ra (μm), with a cut-off length of 0.25 mm. The hardness tester was adapted on a test stand with a glass base plate, in order to conduct the respective measurements. The coated samples were measured after the acid spraying, as well as after the removal of remaining coatings from the concrete surface by using a spatula.

### 3.9. XRD Analysis

The structural phases (i.e., the mineralogical composition) of the samples were detected by the X-ray Diffraction analysis (XRD), using a PW 1840 Phillips diffractometer with CuKa radiation, step size of 0.02°, and step time of 0.4 s, operating at 30 kV and 10 mA. The obtained diagrams were quantified by following the Rietveld methodology, and by using the FullProf Suite Software on the basis of the respective PDF cards numbers #74-2220 (brucite) and #70-0982 (gypsum). The samples were scratched from the surface of the specimens and dried at 60 °C for 24 h. Afterwards the samples were ground and measured.

### 3.10. SEM Analysis

Dried slurries mounted on aluminum stubs with sticky double-side carbon tape. No special treatment was applied to these samples (i.e., measured as dried slurries) and no specific coating needed. Micrographs were obtained by using a Carl Zeiss EVO 50 VP scanning electron microscope (Carl Zeiss SMT Ltd., Cambridge, UK). The examinations were performed at 5 kV accelerating voltage, under variable pressure mode, suitable for non-conductive specimens, and at a pressure of 30 Pa. A variable pressure secondary electron (VPSE) detector was used.

## 4. Results and Discussion

### 4.1. Pull-Off Measurements

The important property of adhesion, regarding the applied coatings on the concrete substrate, was evaluated in this study by using the pull-off technique. Magnesium hydroxide lacks adhesion ability onto a concrete surface, and celluloses were added in order to promote the adhesion. For each examined coating case, two different thicknesses were examined (thin or thick) in order to study the influence of the layer’s thickness on the property of adhesion. According to the aforementioned standards [30,31] the load of failure was recorded in Newtons, and it was used for the calculation of bond strength between the coating and the substrate (expressed in MPa).

The results of tensile bond strength between the coatings and the concrete surface are presented in Table 2 [33], and according to the results, both the thin and the thick layers showed rather similar behavior during adhesion for each coating sample. A small difference can be observed in the case of coating M1m (for both thin and thick layers), however these are the largest values noticed for all the examined coatings.

The series of coatings with the methyl-cellulose additive presented a generally higher tensile bond strength than those with the mixture of both celluloses (methyl-cellulose and carboxy-methyl-cellulose) for the corresponding amounts. M1m showed better adhesion ability than M1mc, although both were synthesized with the total amount of 1% wt. celluloses. Therefore, it can be concluded that the adhesion ability of the examined coatings was reduced with the addition of (even a small portion) carboxy-methyl-cellulose adhesive.

Additionally, it can be observed that the bond strength increased when the amount of cellulose was also increased, notifying that the M1m coating (1% wt. of methyl-cellulose) showed the best adhesion ability onto the concrete substrate, both in thin and thick layers. However, the bond strength values of the M2m and M1mc coatings (0.3 MPa), both for thin and thick layers, were also considered as satisfactory for the desired application on the concrete’s surface. The celluloses can enhance the adhesion of magnesium hydroxide coatings onto concrete; the respective adhesion mechanism is still under investigation, but it is considered possible that hydrogen bonds may be formed, between the hydroxyl groups of the celluloses and the surface of concrete.

### 4.2. Water and Acid Resistance

**Figure 5:**
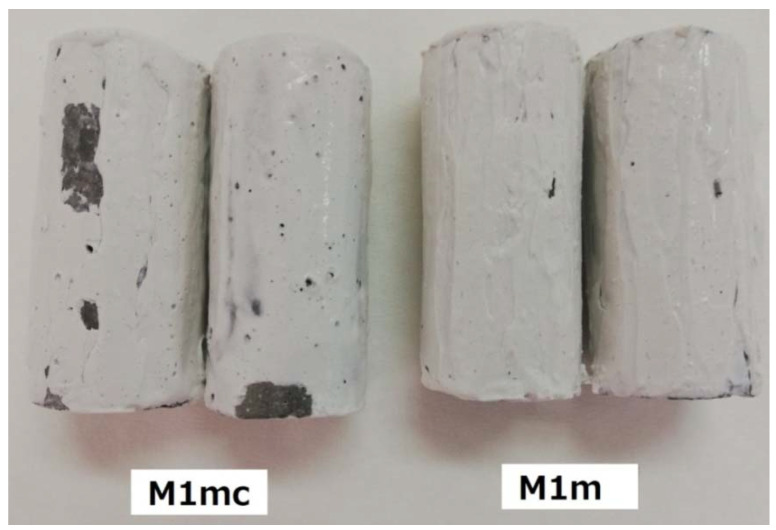
Water resistance of selected coated samples after one-month immersion, and under the influence of slow water circulation (0.05 m/s).

After the period of 1 month, during which the specimens were subjected to the influence of low water flow circulation (0.05 m/s), it was observed that the coatings showing the best adhesion performance were the M1mc and M1m; the M2m sample was next, presenting a slightly lower water resistance. Specifically, the M1m showed better water resistance, when compared to M1mc (Figure 5). The rest of the coating samples showed moderate to poor water resistance. According to these results, the carboxy-methyl-cellulose seemed to present lower adhesion ability, than the methyl-cellulose, as both coatings (i.e., M1m and M1mc) were synthesized with 1% wt. content of the examined celluloses. It can be concluded that the substitution of an amount of methyl-cellulose with an amount of carboxy-methyl-cellulose (i.e., for the M1mc coating) can decrease the adhesion properties of the coating. As far as the rest of the coatings are concerned, a further decrease of the celluloses’ proportions in the coatings (apart from the M2m coating, which was differentiated), cannot offer the desirable adhesion ability. Moreover, the immersion of samples in static water showed quite similar results with those obtained by the low water flow circulation.

However, when all samples were immersed in the acidic solution (H_2_SO_4_ 0.2 M), even under the low circulated experimental conditions, it was noticed that almost all coatings pulled-off almost immediately (that is why the initial concentration of 0.2 M is considered as constant, without any further experimental actions needed to keep the pH constant e.g., by titration), except for the case of coatings M1m and M1mc, which pulled-off after a few minutes. According to the literature, different test methodologies have been used in order to study the degradation of concrete specimens through acid immersion. The standard methods, and the relevant studies, suggest the replacement of the acid solution, after each inspection period, or when the pH value exceeds a pre-selected value [34]. Moreover, titration was used in other studies to retain a stable pH value of the solution used [35]. In this study, the duration of the experiment was very short (due to the coatings’ detachment) and no relevant method for replacing/replenishing the solution was used.

It can be concluded, for both the water and acid resistance tests, that among the three studied amounts of cellulose addition, the highest amount (1% wt. of celluloses) enhanced the adhesion of magnesium hydroxide coatings on the concrete substrate, both in the cases of coatings with methyl-cellulose addition, and in the coatings with the combination of methyl-cellulose and carboxy-methyl-cellulose. The fact that the increased amount of additives can increase the adhesion properties of coatings onto the concrete surface is in agreement with the pull-off tests.

### 4.3. Coating Performance under the Influence of Higher Water Circulation Velocities

**Figure 6:**
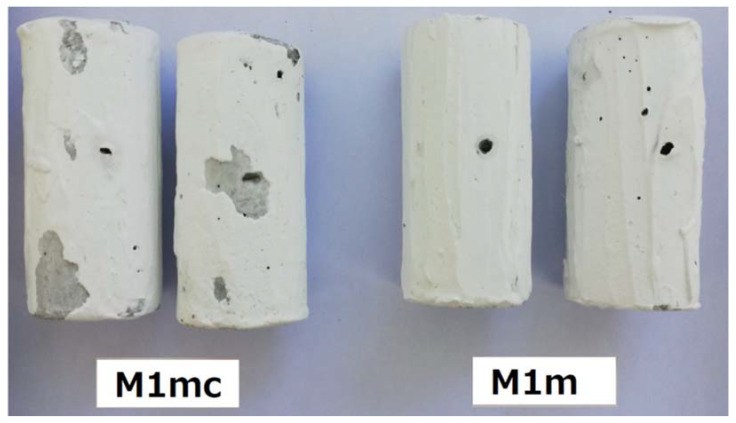
Coating performance (stability) of selected concrete samples under the influence (for 20 min) of high-flow water velocities.

After immersion for 20 min under the influence of higher water circulation velocities, the sample M1m showed still near-perfect coating adhesion performance (only some black spots can be observed), while in the case of M1mc much larger parts of the coating were removed (Figure 6). The large parts may have been detached due to the presence of carboxy-methyl-cellulose, which, as it was explained in Section 4.2, presented smaller adhesion ability, than the methyl-cellulose. These results are in good agreement with the respective pull-off tests.

### 4.4. Acid Spraying

In sewer pipes, the reduction of pH values on the surface of concrete can create the ideal conditions for acidophilic microorganisms to develop, eventually leading to the degradation of the concrete substrate. The produced biogenic sulfuric acid reacts with common calcium compounds of the cement paste, resulting in the formation of gypsum. The coatings suggested in this study can offer corrosion protection in two ways, i.e., (a) by retaining the alkaline surface pH values (and hence, blocking the development of acidophilic microorganisms), and (b) by reacting with (and neutralizing) the biogenic sulfuric acid. The ability of coatings to maintain higher (alkaline) pH values, and resistance to pH (acidic) lowering, was evaluated by using an accelerated method of spraying an appropriate sulfuric acid solution on the surface of the coated concrete specimens. As a result, the sprayed sulfuric acid can react (and neutralize) with the (alkaline) coating, before reacting with the concrete surface and provide protection, whereas the coating is consumed.

The anti-corrosive properties of the examined Mg(OH)_2_-based coatings lie in the neutralization reaction with sulfuric acid. The fundamental reaction between the magnesium hydroxide and the sulfuric acid is given below:

(1) $$\mathrm{Mg}{\left(\mathrm{OH}\right)}_{2}+H_{2}{\mathrm{SO}}_{4}\to {\mathrm{MgSO}}_{4}+2H_{2}O$$

Based on this reaction, 1 mol of magnesium hydroxide reacts with 1 mol of sulfuric acid, producing 1 mol of magnesium sulfate and 2 moles of water. As a result, the concrete surface is protected, because the sulfuric acid cannot reach its surface, as it reacts with the coating. It is important to note that the celluloses were added as adhesion additives and not as reactant agents. However, the effect of cellulose addition amount on the anti-corrosive properties of magnesium hydroxide was studied by using three different cellulose amounts (i.e., 1%, 0.4%, and 0.1% wt. content of celluloses) and two different cellulose combinations (i.e., (a) coatings with methyl-cellulose, and (b) coatings with methyl-cellulose and carboxy-methyl-cellulose).

The coated and uncoated concrete specimens were sprayed with a concentrated sulfuric acid solution (4 M), as described in Section 3.7. The experimental consumption time of the coatings, was expressed as the percentage of the theoretical consumption time (6 days), as shown in Table 3 [33].

Table 3 shows that the thickness (i.e., the used amount) of the coatings can affect the rate of their consumption. By doubling the amount of coating, i.e., when using the thick layer, the increase of “service” duration (i.e., lower consumption rate by the sulfuric acid spray) was almost twice as long. However, it is important to note that each coating was sprayed with an appropriate amount of sulfuric acid corresponding to the respective thickness. The obtained data also shows that all examined specimens can exhibit increased experimental consumption (i.e., neutralization with sulfuric acid) time, when compared with the theoretical value of 6 days.

For the thick coatings, the two specimens containing the highest amount of methyl-cellulose (i.e., M1m and M2m) showed lower consumption rates (i.e., decreased neutralization reactivity by the sulfuric acid addition), while regarding the coatings containing the mixture of both celluloses, the M2mc coating exhibited the slowest consumption rate (and therefore, increased operational time). In both series of thick coatings, those with the smallest cellulose amount (i.e., M3m and M3mc) exhibited the shortest consumption time (i.e., +100%). Regarding the examined cellulose amounts in the prepared coatings, the coatings containing 1% and 0.4% wt. of celluloses were found to last longer than the coatings containing 0.1% wt. of cellulose. However, in order to achieve a more concrete conclusion considering the relation between the consumption time of the coatings and the amount of added cellulose, more experiments with different cellulose amounts should be performed. In contrast, the thin layers of coatings did not present any difference between the coating samples. These results indicate that the coating thickness affects the diffusion of acid through the coating (reaching the concrete surface), and consequently the coatings’ consumption/neutralization rate/time.

**Figure 7:**
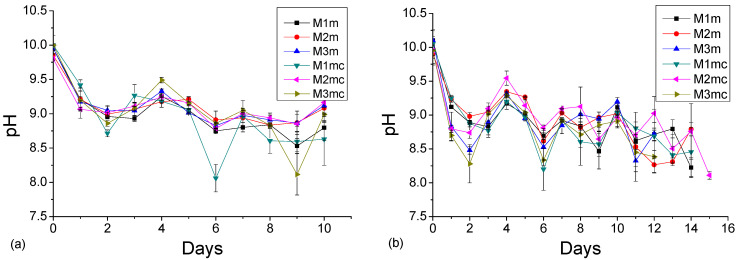
Surface pH values of the concrete specimens sprayed daily with 17% of the reaction’s stoichiometry: (**a**) thin layer, and (**b**) thick layer of coating. The data points and the error bars denote the mean and the standard deviation, respectively.

**Figure 8:**
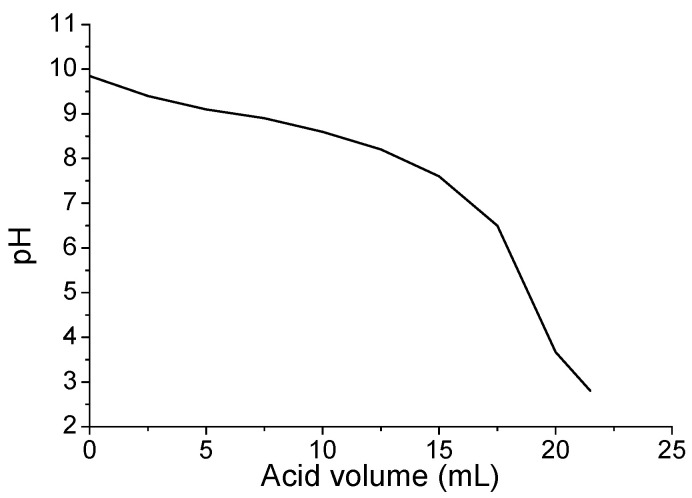
Maximum pH values obtained after 1 min of the addition of each drop (0.5 mL) of a sulfuric acid solution (0.4 M), during the neutralization of Mg(OH)_2_ (MH) (for more information see Appendix B).

Figure 7 presents the daily surface pH values of the specimens sprayed daily with 17% of the respective reaction’s stoichiometry with sulfuric acid, for either (a) the thin, or (b) the thick layer, and for the duration of 10–15 days [33]. The starting pH values for all coatings were around 10. All coatings were found to preserve the surface pH values in the alkaline region, as the surface pH remained above 8 for all the examined cases. This indicates that the coatings were effective in blocking the development of acidophilic microorganisms, which can further produce the biogenic sulfuric acid, leading to the bio-corrosion of concrete surfaces. As aforementioned, the theoretical duration of spraying process was 6 days, but the coating samples lasted more than the theoretical time (Table 3). The thick layer coatings (Figure 7b) were sprayed with a double amount of sulfuric acid daily, resulting to lower surface pH values than the thin layer coatings. Nevertheless, the thick layer coatings exhibited a longer duration of consumption/neutralization (12–15 days) with sulfuric acid than the thin layer coatings (10 days). The surface pH of the coatings exhibited a fluctuation during the accelerated acid spraying test. This buffer-like phenomenon indicated the “alkaline capacity’’ of the examined coatings; the surface pH values were initially decreased after the reaction with sulfuric acid, but after some time they gradually increased again. Magnesium hydroxide, being a weak solid alkaline material, with very low solubility in water, has a buffering effect, i.e., it tends to increase pH values back to 9–10 after neutralizing small amounts of strong acids, such as the sulfuric acid used in the current research. This effect can also be observed in the shape of the respective typical neutralization curve. Figure 8 shows the maximum pH values obtained after 1 min of the addition of each drop (0.5 mL) of a sulfuric acid solution (0.4 M), during the neutralization of the raw material (0.5 g Mg(OH)_2_/100 mL H_2_O). The acid is added at the rate of one drop (0.5 mL) per minute.

**Figure 9:**
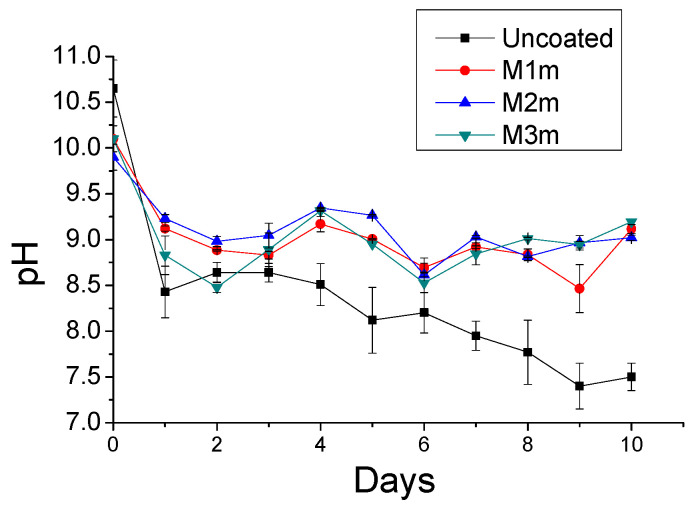
Surface pH values of coated (by methyl-cellulose coatings) and of uncoated concrete specimens, sprayed daily with 17% of the respective reaction’s stoichiometry (using thick layers), during the first 10 days of acid spraying. The data points and the error bars denote the mean and the standard deviation, respectively.

Figure 9 presents the surface pH values for the thick layers of MC coatings, sprayed with 17% of the reaction’s stoichiometry with sulfuric acid, in contrast to the respective uncoated concrete specimens’ surface pH values, during the first 10 days of acid spraying. It can be observed that the uncoated concrete’s surface showed a rapid reduction of surface pH values, and reached the pH value of around 7.5 in a shorter time, i.e., at the 8th days after the acid spraying. This environment may favor the development of acidophilic microorganisms, which can subsequently produce the biogenic sulfuric acid, leading to further concrete degradation. In contrast, the application of the examined coatings can prevent the decrease of pH value that initiates the degradation steps, by maintaining relatively higher alkaline pH values (>8.5).

### 4.5. Roughness and Hardness Properties of Concrete Specimens

**Figure 10:**
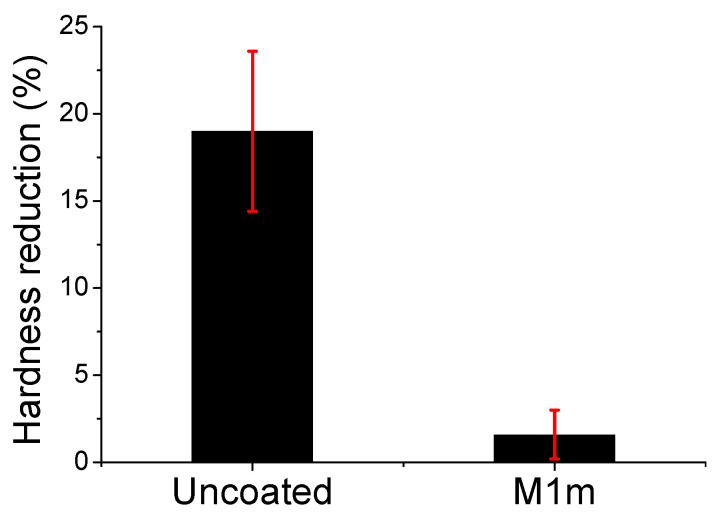
The percentage reduction of hardness in respect to the original concrete hardness of the coated (by M1m) and the uncoated concrete specimens, sprayed with sulfuric acid solution (4 M) by using daily the 17% of the respective reaction’s stoichiometry. The bar charts and the red error bars denote the mean and the standard deviation, respectively.

The fundamental surface properties of hardness and roughness were presented as relative values, in respect to the initial ones of concrete specimens, i.e., those measured before acid spraying. Studies have shown that concrete’s hardness and roughness can change after it is subjected to sulfate attack [24,36]. In that way, the corrosion of concrete or the corrosion protection of concrete can be evaluated through the evaluation of these properties. Figure 10 presents the hardness reduction of the coated and uncoated concrete specimens, sprayed with 4 M sulfuric acid solution (17% of the reaction’s stoichiometry daily). The hardness and roughness measurements conducted on the coated specimens after they were sprayed with sulfuric acid solution, following the procedure described in the Section 3.7. The uncoated concrete specimens were also sprayed with sulfuric acid solution, according to the same procedure, and then the surface hardness and roughness were measured.

The uncoated concrete specimens exhibited about 19% reduction from the initial hardness value of the concrete surface, while the coated specimens showed a substantially lower reduction of hardness. This indicates that the coatings seemed to protect sufficiently the concrete surface and its original properties. The hardness of the concrete surface was generally reduced, due to the formation of gypsum onto the surface, which is produced when the sulfuric acid reacts with the cement paste, neutralizing the alkaline components. In the case of the coated concrete specimens, the coating can react with the sulfuric acid (and hence, the coating thickness was reduced); therefore, it can preserve (protect) the concrete’s initial surface hardness.

**Figure 11:**
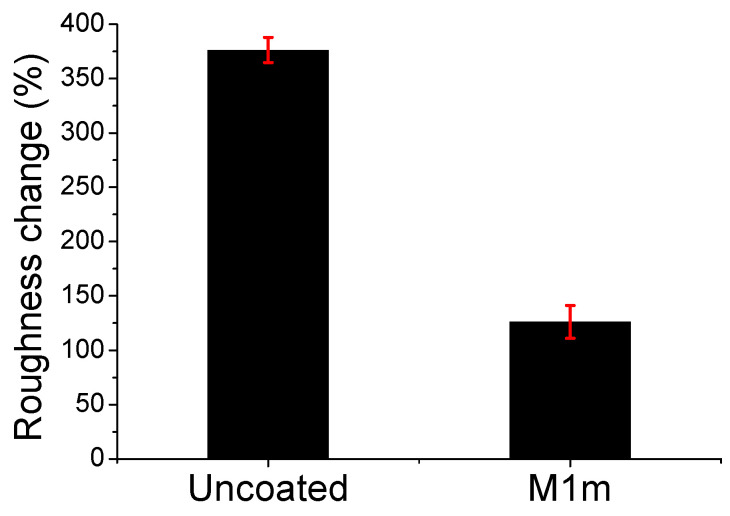
Change of roughness (%) in respect to the original concrete roughness of the coated (by M1m) and the uncoated concrete specimens, sprayed with sulfuric acid solution (4 M) by using daily 17% of the respective reaction’s stoichiometry. The bar charts and the red error bars denote the mean and the standard deviation, respectively.

When the cement paste reacts with sulfuric acid and gypsum is formed, the inert aggregates content is left exposed on the surface, or they can even remove from the concrete substrate when they become fully exposed. Some researchers showed that chemical attack can lead to an increase of concrete surface roughness [37]. The respective results showed in Figure 11 present a large increase of surface roughness for the uncoated concrete specimens, which were sprayed with sulfuric acid, in relation to the original roughness. On the contrary, the coated specimens’ (protected) surface exhibited substantially smaller increase in roughness, than that of the uncoated samples.

### 4.6. XRD Analysis

**Figure 12:**
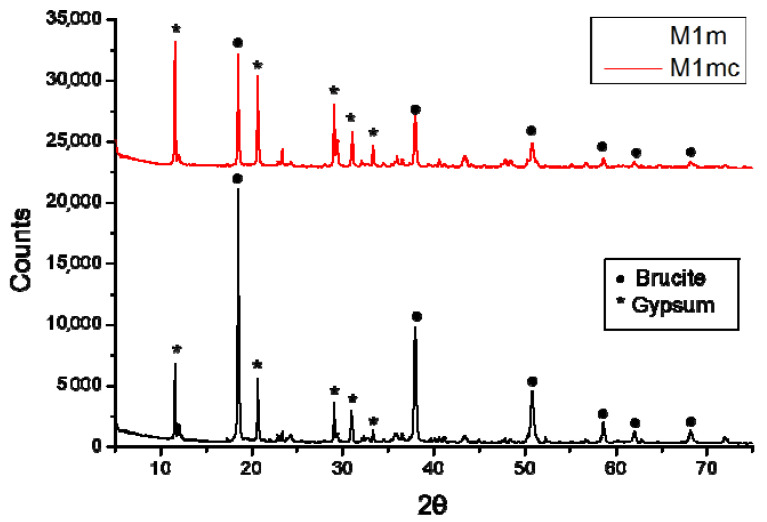
XRD overlay diffractograms of the coatings, M1m and M1mc, after the effect of acid spraying, with sulfuric acid solution (4 M) for 10 days (using daily the 17% of the respective reaction’s stoichiometry).

The coatings (and the formed by-products) were scratched/removed from the concrete specimens after they were sprayed with 4 M sulfuric acid solution, using the daily stoichiometry 17% of the respective reaction, according to the procedure described in Section 3.7, and were analyzed by XRD. Figure 12 presents the XRD overlay diffractograms for the coatings M1m and M1mc. The different intensity of gypsum and brucite peaks between the two coatings can observed. The M1m coating presented higher intensity brucite peaks, while the M1mc coating presented lower intensity brucite peaks. In contrast, the intensity of gypsum peaks is higher in the M1mc coating’s diffractogram. The XRD results were further elaborated with the use of the Rietveld refinement method, and applying FullProf software in order to evaluate/quantify the content (as weight percentage) of the existing crystalline phases (initial and formed by-products).

**Figure 13:**
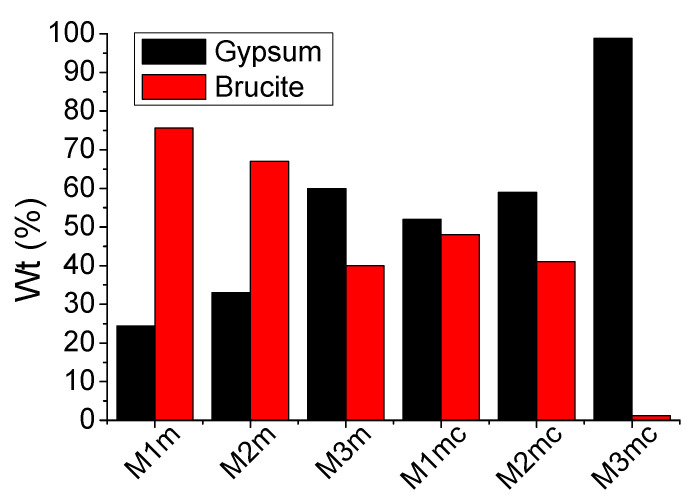
The formation of gypsum and brucite (% weight) of all the examined coatings after acid spraying with sulfuric acid solution (4 M) for 10 days, using daily the 17% of the respective reaction’s stoichiometry.

Table 2 and gypsum (CaSO_4_) formation for all the examined coatings, are presented in Figure 13. Brucite and gypsum percentages were found to vary when different coatings were applied for corrosion protection. Among all the examined coatings, those containing 1% and 0.4% wt. of cellulose (i.e., M1m and M2m) consisted of more brucite than gypsum, while the other coatings presented the opposite results.

The series of coatings containing both celluloses (mixture) presented more gypsum formation, than the coatings containing only methyl-cellulose, when using similar coating amounts. Apparently, the addition of carboxymethyl-cellulose enhanced the formation of gypsum. For example, despite the same consumption time, the M1mc coating exhibited a lower brucite amount formation and a higher gypsum amount than the M1m coating. This result indicates that the optically observed coating (i.e., that remaining on the concrete surface after acid spraying) which was collected for the M1mc coating, provoked the increased gypsum formation, with respect to the brucite amount. A possible explanation is that the inner morphology of the M1mc coating could permit the diffusion of sulfuric acid through the coating mass; hence, reaching the concrete surface and leading to the formation of gypsum on the coating–concrete interface. Therefore, and despite the fact that both coatings presented the same consumption time, they exhibited different corrosion protection abilities.

The M3mc coating presented the greatest decrease in the brucite phase among all the examined coatings, whereas the M3m exhibited the greatest gypsum formation of the coatings containing methyl-cellulose. These results showed that the amount of added cellulose also affects the consumption of coatings, as well as the formation of corrosion (by-)products [33]. According to the obtained results, the presence of methyl-cellulose seemed to moderate the extended activity of brucite, which resulted in the longest protection capacity, whereas the addition of carboxy-methyl-cellulose (even a small portion) allowed the fast reaction of brucite with sulfuric acid (and the respective fast consumption of coating), leaving the concrete surface rather exposed (as gypsum was also created).

### 4.7. SEM Analysis

**Figure 14:**
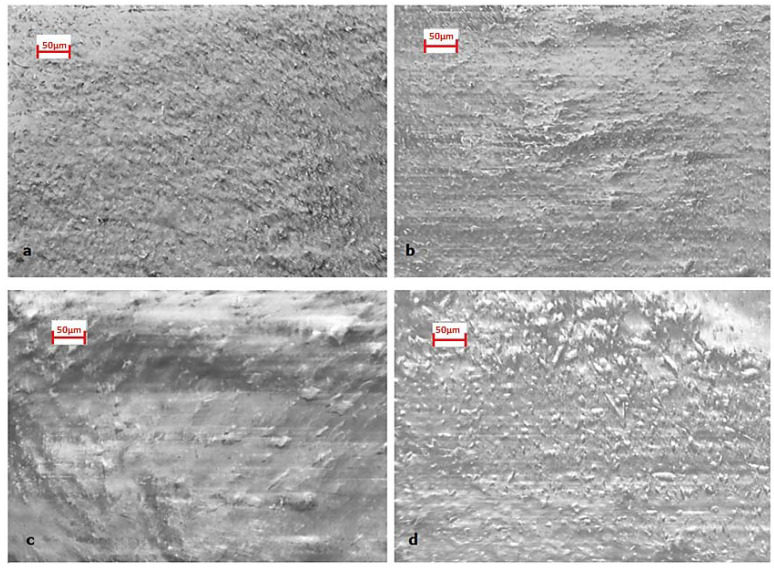
Comparison of SEM micrographs, regarding the surface of coatings: (**a**) M3mc, (**b**) M3m, (**c**) M1mc, and (**d**) M1m.

The micrographs of four selected dried coatings are presented in Figure 14. The coatings with 1% and 0.1% wt. of cellulose mixture are compared, because the coatings with 1% of cellulose content exhibited the optimal results under the studied conditions, and the coatings with 0.1% of cellulose content exhibited the least satisfactory results. The magnesium hydroxide slurries, containing methyl-cellulose as adhesive at higher concentrations, i.e., 1% wt. of cellulose (e.g., M1m), as well as 1% wt. of cellulose mixture (methyl-cellulose and carboxy-methyl-cellulose, e.g., M1mc) showed a rather smooth surface. In comparison, the samples M3m and M3mc (0.1% wt. content of celluloses), presented a rougher surface, based on the SEM micrographs. Moreover, the M1mc coating surface exhibited the smoothest surface of all coatings, with larger grains formed on its surface. The coating surface of M1m seemed to develop larger particles than the M3m coating, while the M3m exhibited smaller and denser particles. M3mc also presented denser and smaller surface particles than the M1mc sample.

The observed different surface characteristics may affect the reaction between the coatings and the sulfuric acid. The addition of celluloses seemed to lead to the creation of Mg(OH)_2_ aggregates, which may slow down its rapid reaction with sulfuric acid. The formation of large aggregates (i.e., M1m and M1mc) may lead to a smaller exposed surface of magnesium hydroxide that is available to react, than when smaller aggregates are formed (i.e., M3m and M3mc) [38]. This fact explains the longer experimental consumption time for the coatings M1m and M1mc, in respect to the M3m and M3mc samples for the thick layers applied (Table 3).

However, the aggregates of the M1mc coating presented a different morphology than the M1m coating. Probably, this morphology affected the reaction mechanism of the M1mc coating, leading to increased gypsum formation, according to the aforementioned XRD quantitative results. As explained in Section 4.6, the diffusion of sulfuric acid through this coating could have led to the corrosion of the concrete surface under the coating, without the previous consumption of the protective coating. In that way, the duration of the M1m and M1mc coatings’ consumption is the same (Table 3), but in the case of M1mc coating the formed gypsum was increased, as compared with brucite formation. Moreover, the morphology of the inner layers of the coatings should be further examined, to explain better the coating’s behavior.

## 5. Conclusions

In this research, six different Mg(OH)_2_-based coatings were examined, regarding their application onto the surface of concrete sewer pipes, acting as potential bio-corrosion inhibitors. Their adhesion ability was found to depend on the amount of cellulose addition. The magnesium hydroxide-based coatings seemed to protect the concrete surface longer than the respective theoretically calculated consumption time, considering the neutralization reactions with sulfuric acid, and seemed to maintain the surface pH towards alkaline values; therefore, inhibiting the action of acid-producing bacteria.

Among the various coatings, containing methyl-cellulose, that with 1% wt. content of celluloses showed better adhesion ability, water resistance, and acid attack resistance properties. According to the quantitative evaluation of the XRD results, the coatings with 1% and 0.4% wt. of methyl-cellulose (i.e., M1m and M2m) exhibited the lowest formation of gypsum, after spraying with sulfuric acid between all examined coatings, indicating better corrosion protection. Furthermore, the corrosion protection ability of the studied coatings was supported by the respective hardness and roughness results of concrete’s surface, in comparison with the uncoated (but sprayed) concrete specimens.

SEM micrographs showed the different surface roughness among the examined coatings. In addition, the coatings with the higher cellulose content exhibited the formation of aggregates that may affect the reaction (neutralization) between magnesium hydroxide and sulfuric acid. These aggregates may block the (otherwise) intense reaction activity, and could lead to the decrease of the coating’s relevant consumption rate (and, hence, to the increase of operational time).

Regarding future research studies, the authors will examine the effectiveness of the most promising applied protective coatings, but used to protect a regular size concrete pipe under realistic sewer network conditions. Moreover, raw materials, also based upon magnesium hydroxide but with different physical properties, will be studied, in order to evaluate the effect of these properties on the protection ability for concrete corrosion.

## Figures and Tables

**Table 1:** Mg(OH)_2_ powder composition (%) used for the coatings’ preparation.

Material	MgO	SiO_2_	CaO	Fe_2_O_3_	Al_2_O_3_	SO_3_	LOI
Mg(OH)_2_	62.81	4.25	2.46	0.25	0.1	0.02	30.11

**Table 2:** Pull-off results of the coatings for the examined thick and thin layers, with the respective standard deviation (SD) values [33].

Acronym	Thick Layer		Thin Layer	
Coating	fh (MPa)	SD	fh (MPa)	SD
M1m	0.6	0.033	0.5	0.085
M2m	0.3	0.002	0.3	0.057
M3m	0.1	0.033	0.1	0.015
M1mc	0.3	0.047	0.3	0.018
M2mc	0.1	0.004	0.1	0.001
M3mc	0.1	0.036	0.1	0.007

**Table 3:** The experimental consumption time expressed as percentage (and in comparison with) of the theoretical consumption time for the examined coated samples.

Basis: Theoretical 6 days		
Coating	Thin Layer	Thick Layer
M1m	+67	+133
M2m	+67	+133
M3m	+67	+100
M1mc	+67	+133
M2mc	+67	+150
M3mc	+67	+100

**Figure A1:**
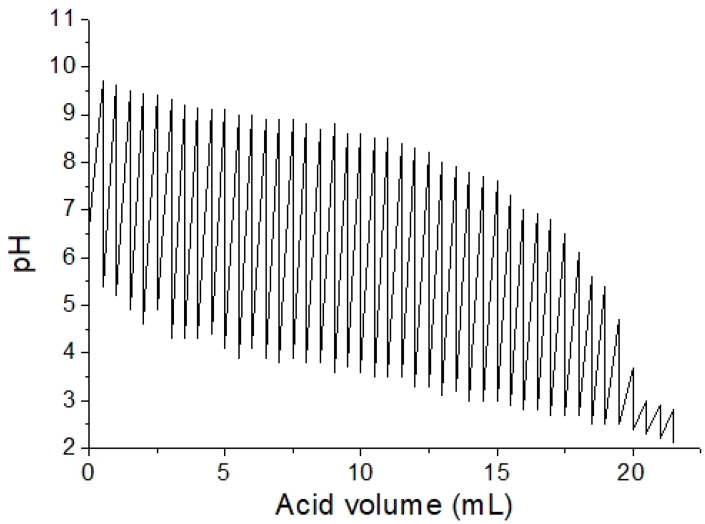
Neutralization curve of Mg(OH)_2_ (MH) with a sulfuric acid solution (0.4 M), adding a 0.5 mL drop of acid per minute.

## Appendix A

Stoichiometry calculations:

The raw material used for the preparation of coatings is mainly composed of Mg(OH)_2_. Magnesium hydroxide and calcium hydroxide (present as a smaller percentage in the raw material) are the main components of the examined coatings, which can react (neutralization reactions) with the sulfuric acid solution. The daily consumption of any specific amount of coating depends on the sulfuric acid amount that is sprayed on the concrete specimen.

The neutralization reaction between magnesium hydroxide and sulfuric acid is:

Mg(OH)_2_ + H_2_SO_4_ → MgSO_4_ + 2H_2_O (A1)

While the relevant reaction between calcium hydroxide and sulfuric acid is:

Ca(OH)_2_ + H_2_SO_4_ → CaSO_4_ + 2H_2_O (A2)

According to these reactions 1 mole of Mg(OH)_2_ or 1 mole of Ca(OH)_2_ needs 1 mole of H_2_SO_4_ each.

In order to perform an accelerated corrosion test, 6 days was selected as the duration of the experiment. During this time, the sulfuric acid that is sprayed should correspond to the full consumption of the coating (i.e., for 100% of the respective reaction’s stoichiometry). According to this, the daily addition of sulfuric acid should be 17% of the theoretical (=100%/6 days).

Each concrete specimen was coated/sprayed with 5 g of slurry containing 57.5% wt. of solids. The coatings’ mass that theoretically reacts with the sulfuric acid was expressed, based on the respective magnesium and calcium content. According to Table 1, the raw material was composed from 62.81% MgO and 2.46% CaO. The moles of MgO and CaO contained in the 5 g of slurry were calculated, added, and resulted to the sum of moles that can potentially react (and be neutralized) with the sulfuric acid, considering 100% stoichiometry of the overall reaction.

Based on the aforementioned reactions, the total moles of the sulfuric acid needed to complete a 100% stoichiometry of the neutralization can be calculated (Mg(OH)_2_ moles + Ca(OH)_2_ moles = H_2_SO_4_ moles). Therefore, the moles of sulfuric acid that correspond to a daily reaction stoichiometry of 17% can be calculated.

A concentrated sulfuric acid solution of 4M was selected for the experiments. The limitation of spraying time, due to the use of a handheld spraying device, led to the selection of the initially high concentrated sulfuric acid solution, offering the restricted time duration of the spraying experiment. The concentration of this solution was used to convert the number of moles to the volume of sulfuric acid that was needed to achieve a 17% stoichiometry of the (daily) neutralization reactions, considering the magnesium and calcium content for the respective amount of used/applied coating.
